# Molecular and phenotypic characteristics influencing the degree of cytoreduction in high‐grade serous ovarian carcinomas

**DOI:** 10.1002/cam4.6085

**Published:** 2023-05-16

**Authors:** Cecilie Fredvik Torkildsen, Liv Cecilie Vestrheim Thomsen, Ragnar Kvie Sande, Camilla Krakstad, Ingunn Stefansson, Eva Karin Lamark, Stian Knappskog, Line Bjørge

**Affiliations:** ^1^ Centre for Cancer Biomarkers, Department of Clinical Science University of Bergen Bergen Norway; ^2^ Department of Obstetrics and Gynecology Stavanger University Hospital Stavanger Norway; ^3^ Department of Obstetrics and Gynecology Haukeland University Hospital Bergen Norway; ^4^ Department of Clinical Science University of Bergen Bergen Norway; ^5^ Department of Pathology Haukeland University Hospital Bergen Norway; ^6^ Department of Oncology Haukeland University Hospital Bergen Norway

**Keywords:** cancer genetics, clinical observations, gynaecological oncology, mutations, risk model, surgery

## Abstract

**Background:**

High‐grade serous ovarian carcinoma (HGSOC) is the deadliest ovarian cancer subtype, and survival relates to initial cytoreductive surgical treatment. The existing tools for surgical outcome prediction remain inadequate for anticipating the outcomes of the complex relationship between tumour biology, clinical phenotypes, co‐morbidity and surgical skills. In this genotype–phenotype association study, we combine phenotypic markers with targeted DNA sequencing to discover novel biomarkers to guide the surgical management of primary HGSOC.

**Methods:**

Primary tumour tissue samples (*n* = 97) and matched blood from a phenotypically well‐characterised treatment‐naïve HGSOC patient cohort were analysed by targeted massive parallel DNA sequencing (next generation sequencing [NGS]) of a panel of 360 cancer‐related genes. Association analyses were performed on phenotypic traits related to complete cytoreductive surgery, while logistic regression analysis was applied for the predictive model.

**Results:**

The positive influence of complete cytoreductive surgery (R0) on overall survival was confirmed (*p* = 0.003). Before surgery, low volumes of ascitic fluid, lower CA125 levels, higher platelet counts and relatively lower clinical stage at diagnosis were all indicators, alone and combined, for complete cytoreduction (R0). Mutations in either the chromatin remodelling SWI_SNF (*p* = 0.036) pathway or the histone H3K4 methylation pathway (*p* = 0.034) correlated with R0. The R0 group also demonstrated higher tumour mutational burden levels (*p* = 0.028). A predictive model was developed by combining two phenotypes and the mutational status of five genes and one genetic pathway, enabling the prediction of surgical outcomes in 87.6% of the cases in this cohort.

**Conclusion:**

Inclusion of molecular biomarkers adds value to the pre‐operative stratification of HGSOC patients. A potential preoperative risk stratification model combining phenotypic traits and single‐gene mutational status is suggested, but the set‐up needs to be validated in larger cohorts.

## Graphical abstract

New biomarkers to guide surgical management of primary ovarian cancer are warranted. By adding data from DNA sequencing to phenotypic traits we demonstrate that molecular biomarkers adds value to the pre‐operative stratification process. A potential preoperative risk stratification model that might increase the accuracy is suggested.

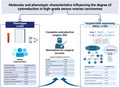

## 1 INTRODUCTION

Despite recent advances in treatment possibilities, epithelial ovarian cancer (EOC) is still the most lethal gynaecological malignancy. The established first‐line standard of care treatment for patients with advanced EOC is a combination of surgery and chemotherapy, but the optimal timing of the surgery is controversial.^1^ High‐grade serous ovarian carcinoma (HGSOC) is the most common histological subtype of EOC and is characterised by rapid direct spread with transcoelomic dissemination of malignant cells throughout the abdominal cavity. The characteristic vague disease‐associated symptoms appear because of disseminated disease; consequently, more than 60% of the patients have advanced disease (International Federation of Gynaecology and Obstetrics [FIGO] stages III‐IV) at diagnosis. The prognosis is poor, with a 5‐year overall survival rate of 51.1%.^2^

In addition to the cancer stage at diagnosis, the degree of cytoreductive surgery in the primary setting matters, and together, they represent the most important prognostic markers for HGSOC. The positive effect of surgery, even for patients with metastatic disease, has been demonstrated in multiple trials, with the best impact on overall survival (OS) when there is no residual disease after surgery (R0). Consequently, surgical treatment has been the subject to a vast evolution in which ultra‐radical techniques have been developed to increase the rate of complete cytoreduction.^3^, ^4^, ^5^ However, all improvements come at a price. The morbidity and mortality are not negligible, the procedures demand longer operating theatre times, and infrastructure and financial resources can be a challenge.^6^ An important additional issue is that despite these surgical improvements, some patients still end up with residual tumour tissue.^7^, ^8^, ^9^, ^10^ For the latter group, a preoperative decision leading to neoadjuvant chemotherapy (NACT) and, thereafter, interval surgery would probably have been preferable.^11^ While we are waiting for the results of the TRUST trial to mature, the optimal stratification towards primary cytoreductive surgery or NACT remains unresolved.^12^

Today, preoperative assessment is managed with different imaging modalities and laparoscopic and phenotypic risk scores alone or combined. Treatment institutions have adapted different algorithms depending on local practices and personal experiences. None of these stratification methods has demonstrated excellence, and they poorly consider the biological diversity that exists, with the presence of at least five different histological subtypes of HGSOC. The ideal primary treatment sequence seems to depend on multiple factors beyond the metastatic pattern and the established different phenotypic traits.

During the last decade, phenotypic and molecular biomarkers have been gradually introduced to direct maintenance therapy with inhibitors of poly‐ADP‐ribose polymerase (PARP)^13^, ^14^ and anti‐angiogenetic agents^15^, ^16^, ^17^ for patients with advanced disease.^18^, ^19^, ^20^ The tumour biological characteristics of HGSOC have been explored, resulting in the identification of at least four molecular subtypes.^21^, ^22^ Differences in mutational status could further predict how specific clinical phenotypic patterns can indirectly cause unresectable tumours, but the available data have not demonstrated consistent significant phenotype–genotype associations, the sample sizes have been small and the point mutations have been few. None of these methods are mature or promising enough to be implemented as diagnostic tools.^22^, ^23^, ^24^, ^25^ Seven copy number signatures have been described as indicators of early relapse, survival and platinum resistance, and this biomarker seems to be robust. However, as the data are generated using whole genome sequencing and extensive bioinformatics,^26^ the analysis is currently still too time‐consuming and costly to be used in the preoperative decision‐making process at most treatment centres.^27^, ^28^

Complete cytoreductive surgery is a crucial indicator for survival and depends on the phenotypic disease pattern, co‐morbidity and biological characteristics. To discover novel biomarkers for the preoperative prediction of surgical outcomes, we performed an extensive phenotypic characterisation of an HGSOC cohort and combined the information with molecular data from targeted analyses of a panel of cancer related genes.

## 2 MATERIALS AND METHODS

### 2.1 Patient characteristics

**FIGURE 1:**
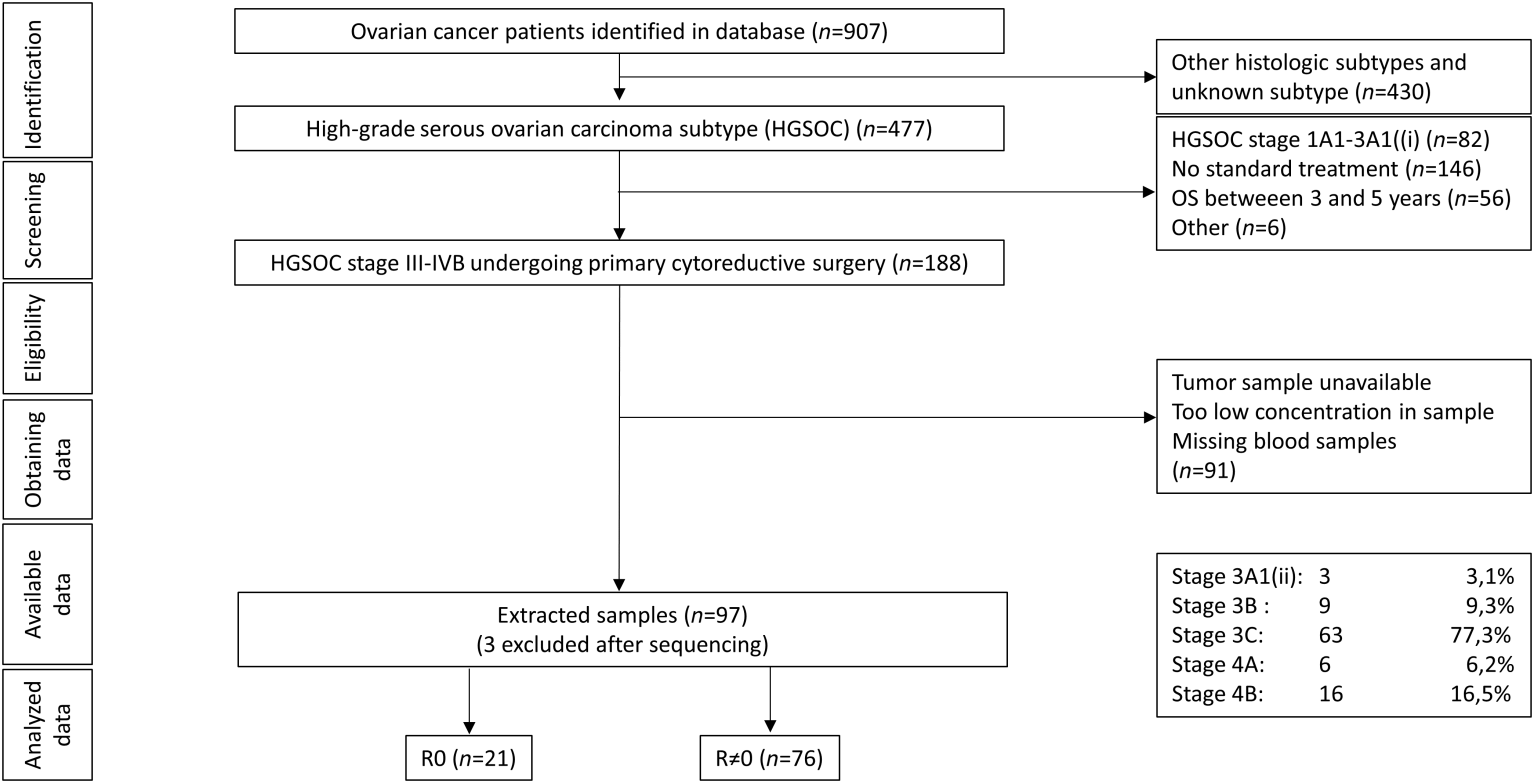
Flow chart depicting the selection from the biobank of patients for inclusion. The inclusion criteria included a confirmed HGSOC diagnosis and the fact that the patients underwent standardised treatment defined as primary cytoreductive surgery and postoperative chemotherapy. The FIGO 2014 classification was used for cancer staging. OS: overall survival; R0: no residual tumour tissue after primary cytoreductive surgery; R ≠ 0: any residual tumour tissue (irrespective of size) after primary cytoreductive surgery; FIGO: the International Federation of Gynaecology and Obstetrics classification of malignant ovarian tumours from 2014.

The original cohort of 905 patients represents a collection of all patients diagnosed with EOC at Haukeland University Hospital, Bergen, Norway during the period of August 2001–January 2017 (Figure 1). From this cohort, 97 patients with advanced HGSOC (FIGO stage 3 or 4) who underwent primary cytoreductive surgery followed by carboplatin‐based chemotherapy were selected for the present study. During the inclusion period, PARP inhibitors were not implemented as part of the recommended primary setting treatment regime. In this cohort, a PARP inhibitor was used by four patients as part of the treatment of recurrent disease. Patients tested for germline or somatic BRCA mutations as a part of the clinical follow‐up were included in the analysis. These patients have received information about their mutation status and genetic counselling (*n* = 34, 35%). However, as Norwegian regulations prohibit BRCA testing of patients without a specific informed consent we cannot assess the BRCA mutation status for the remaining cohort. The patient demographic data, phenotypic characteristics and treatment were available for analysis (Table S1). Based on a thorough review of the surgical and histological report, the patient group was subdivided into two main categories based on surgical outcomes: Patients with no visual residual tumour tissue after surgery (R = 0) and those who had residual tumour tissues after surgery (R ≠ 0). To enable further relevant analysis, the largest residual tumour lesion was registered: Radical (R = 0, no visual residual tumour) and the R ≠ 0 patients were classified for optimal (R = 1, largest lesion ≤1 cm) and suboptimal (R = 2, largest lesion >1 cm) surgery. We defined the term “clinical stage” like Sobin et al. Clinical stage is a combined parameter which combines the level of disease dissemination according to pre‐treatment imaging, and results from other diagnostic medical procedures performed before the cancer treatment was initiated. The disease dissemination was evaluated according to the FIGO 2014 scoring system. The ascitic fluid volume was accurately estimated when a preoperative or a per‐operative drainage was performed. In the remaining patients, the volume was estimated based on the surgeons' description combined with preoperative radiological findings. To avoid inter‐individual variation in descriptive terms like “moderate” and “small”, we required additional compatible data from imaging in order to categorise the amount into <500 mL or >500 mL. Cases without any description of ascitic fluid volume were categorised as “unknown” (*n* = 2). Altogether, nine patients underwent secondary surgery; four patients underwent a comprehensive secondary cytoreductive surgical procedure, while in five patients only solitary lymph nodes were removed. However, these procedures were performed according to the surgeon's preference and were not based on standardised approaches. Secondary surgery was therefore not included in the analyses.

### 2.2 Tumour tissue collection and processing

After collection at the time of primary diagnosis, the samples were immediately snap‐frozen in liquid nitrogen. Only tumour biopsies from ovaries were included. The tumour content was assessed in ethanol‐fixed and haematoxylin‐ and eosin‐stained sections and validated at Haukeland University Hospital's Department of Pathology by a skilled pathologist. The minimum tumour content cut‐off for inclusion was set at 50%, and the majority of the samples had a tumour purity of more than 70% (*n* = 72). DNA was isolated from the fresh frozen samples using Qiagen Allprep® DNA/RNA Mini Kit (Qiagen). The procedure was performed according to the manufacturer's recommendations. DNA quantification was performed using both a NanoDrop M‐1000 spectrophotometer (Thermo Fisher Scientific) and a Qubit Fluorometer (Thermo Fisher Scientific).

### 2.3 Blood sampling and processing

Fresh frozen whole blood samples from EDTA tubes collected at the time of diagnosis were used. Qiagen's DNeasy® Blood and Tissue Kit was used for isolation of DNA from whole blood, according to the manufacturer's recommendations.

### 2.4 Sequencing and mutation calling

Sequencing was performed as previously described.^29^ In brief, a total of 1000 ng of dsDNA was fragmented into pieces of 150–200 bp using the Covaris® M220 Focused‐ultrasonicator™ (Covaris Woburn). The quality was assessed with the Bioanalyzer DNA 1000 assay. Library preparation was performed using the Agilent SureSelect XT‐kit (Agilent Technologies). We used an in‐house panel covering 360 cancer‐related genes. The panel is previously described.^30^ In brief, it consists of a selection of genes (360) that are known or suspected to play a role in cancer. The design included +/− 10 nucleotides at exon–intron borders to cover potential splice site mutations. End‐repair and readenylation were performed to facilitate ligation between the adaptors and the sheared DNA fragment.

The samples were ligated with paired end adaptors and amplified using KAPA HiFi polymerase before being quantified and quality controlled. The samples were then lyophilized using a vacuum centrifuge resuspended into smaller volumes to normalise the concentrations and hybridised to the SureSelect capture library. After hybridisation, the DNA was index‐tagged using KAPA HiFi polymerase before they were pooled and heat‐denatured into single‐stranded DNA before the library mix was loaded onto the reagent cartridge in the designated reservoir added to the flow cell. We used a 150‐cycle PE cartridge with 76 reads for cycles 1 and 2. One read was 150 pb of DNA sequence. Sequencing depth ranged from 200 to 300 x. All samples were run on a MiSeq instrument (Illumina). Alignment and mutation calling was performed using the Dragen server software (v.3.8), with additional post‐processing filters removing variants with VAF <0.05 and variants outside of protein coding regions and/or essential splice sites.

### 2.5 Pathway analysis

In addition to assessing associations between clinical features/phenotypes and alterations in single genes, we also measured associations with functional pathways. Different mutations shown to cause a comparable functional outcome can be assembled into pathways using functional enrichment analysis. For this purpose, we predefined a total of 19 pathways, including the Fanconi anaemia homologous recombination repair pathway, covering HRD genes (Table S3). In addition, we performed extended HRD analysis which have been previously described.^29^

### 2.6 Statistical analyses

We performed the Shapiro–Wilk test to assess the normality assumption. Non‐normally distributed data were log‐transformed. The Mann–Whitney U, and Kruskal–Wallis tests were applied to investigate the potential differences in continuous variables (age, weight, blood values, TMB) between groups (R ≠ 0 vs. R = 0, disease stage, mutation status, ascitic fluid >500 mL, symptoms and ECOG status). The categorical variables were analysed using Fisher's exact test. Univariate survival analysis was performed using the Kaplan–Meier method, and the patients were compared using the log‐rank test. Multivariate survival analyses were performed in a one‐step fashion using the Cox proportional hazards regression model. Known clinical predictors of survival, such as age and disease stage, were added as categorical covariates. TMB was estimated as number of mutations per megabase. All *p*‐values were reported as two‐sided, and *p*‐values <0.05 were considered significant. All statistical analyses were performed using the SPSS 26.0 software package (SPSS INC.).

## 3 RESULTS

### 3.1 Phenotypic characteristics for complete cytoreductive surgery (R0)

In a selected group of patients (*n* = 97), we explored the phenotypic characteristics of patients undergoing complete cytoreductive surgery. The main demographic and clinical data are summarised in Table 1. The data for the entire cohort is presented in Table S1.

**TABLE 1:** Demographic data of the participants (*n* = 97) in the study.

Phenotype/patient characteristics	Numeric/percentages (range)
Age in years (mean, 95% CI) (*n*)	64 (62–66.3)
Stage (FIGO 2014)	
Stage 3 (%)	77.3
Stage 4 (%)	22.7
Neoadjuvant chemotherapy (%)	0
Complete cytoreductive surgery (%)	22.7
BMI (kg/m^2^) at diagnosis (mean, 95% CI) (*n*)	25.3 (24.4–26.2)
CA125 (kU/L) at diagnosis (mean, 95% CI) (*n*)	1260 (929–1593)
ASA score (mean, 95% CI) (*n*)	2 (2.1–2.3)
ECOG score (mean, 95% CI) (*n*)	1 (0.6–0.9)
Surgical complexity score (mean, 95% CI) (*n*)	4 (3.2–4.0)
Clavien Dindo score (mean, 95% CI) (*n*)	2 (1.5–2.0)
Peroperative ascitic fluid, mL (mean, 95% CI) (*n*)	1614 (1220–2007)
CA125(U/mL) after surgery (mean, 95% CI) (*n*)	778 (361–1195)
Postoperative chemotherapy (%)	97.1
Recurrence (%)	88
PFS (mean (months), 95% CI) (*n*)	25.8 (19–33)
OS, (mean (months), 95% CI) (*n*)	49.6 (41–59)

*Note*: FIGO 2014; The International Federation of Gynaecology and Obstetrics classification of malignant ovarian tumours from 2014; An extended version is presented in Table S2.

Abbreviations: ASA, American Society of Anesthesiologists Physical Status Classification System; BMI, Body Mass Index; CA125, Cancer antigen 125; ECOG, Eastern Cooperative Oncology Group performance status; PFS, Progression‐free survival; OS, Overall survival.

Associations were found between surgical outcome and increased volume of ascitic fluid (*p* = 0.008), stage (*p* = 0.014) and CA125 levels at diagnosis (*p* = 0.022), postoperative CA125 levels (*p* < 0.001), lower platelet count at diagnosis (*p* = 0.019), longer time to adjuvant treatment start after surgery (*p* = 0.026), prolonged overall survival (*p* = 0.009) and progression‐free survival (*p* = 0.006) and more advanced disease based on preoperative assessments (*p* = 0.006). Among the categorical variables, we found that ascites <500 mL (*p* = 0.002) was an individual significant predictor for R0 (Table 2, Table S2). However, no association was found between complete radical surgery and age, body mass index (BMI), American Society of Anesthesiologists Physical Status Classification System (ASA) score or Eastern Cooperative Oncology Group performance status (ECOG) score. The survival analysis demonstrated an increased overall survival in the complete surgery group (Figure 4).

**TABLE 2:** Phenotypic markers significantly associated (*p* < 0.05) with complete cytoreductive surgery (R0).

Variable	*p*‐value R0 vs R ≠ 0
Categorical survival: <3 years/>5 years	0.011
Ascitic fluid: <500 mL	0.002
Clinical stage^a^	0.006
Surgical complexity score	0.000
Rubricated response evaluation^b^	0.000
Total volume ascites	0.008
Stage of disease (FIGO)	0.014
CA125 value at diagnosis	0.022
Platelet numbers/mL venous blood at diagnosis	0.019
Time from surgery to chemotherapy	0.026
CA125 before initiation of chemotherapy	0.000
Progression‐free survival	0.006
Overall survival	0.009

*Note*: R0: Complete cytoreductive surgery, R ≠ 0: Residual tumour tissue after surgery; FIGO: International Federation of Gynaecology and Obstetrics classification of malignant ovarian tumours; CA125: Cancer antigen 125.

^a^ Disease stage based on preoperative imaging and medical procedures before treatment is initiated. Clinical staging was performed according to the FIGO 2014 scoring system.

^b^ Response Evaluation Criteria in Solid Tumours (RECIST criteria), modified for ovarian cancer patients.

### 3.2 Mutation profile

**FIGURE 2:**
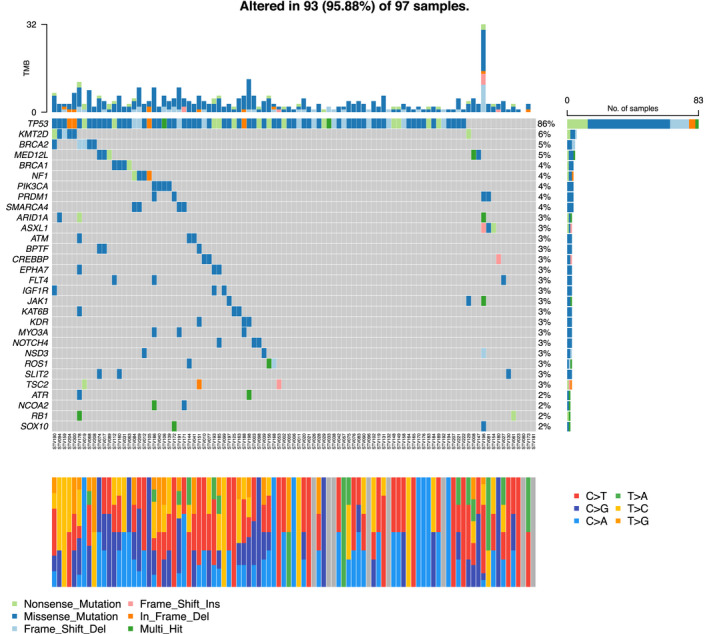
An oncoplot mutation list of all mutations detected by targeted DNA sequencing (360 gene panels) of ovarian cancer patients (*n* = 97). Genes are altered in 93 (95%) of 97 samples. Genes are listed on the left. Bars on the right indicate the prevalence of each mutation among the tumours analysed. Mutations are colour‐coded based on the type of mutation detected. Patient IDs are given below the columns, and each column represents one tumour/patient. At the top, the tumour mutational burden (TMB) is demonstrated. TMB was calculated by adding all missense, insertions/deletions and frameshift variants within the tumour sample and dividing by the total size of the panel. In the stacked bar plot, the bars are coloured according to the type of mutation discovered: C > T; red, C > G; dark blue, C > A; light blue, T > A; green, T > C; yellow, T > G orange (see also Figure S1). The different *TP53* mutations are presented in Figure S2.

**FIGURE 3:**
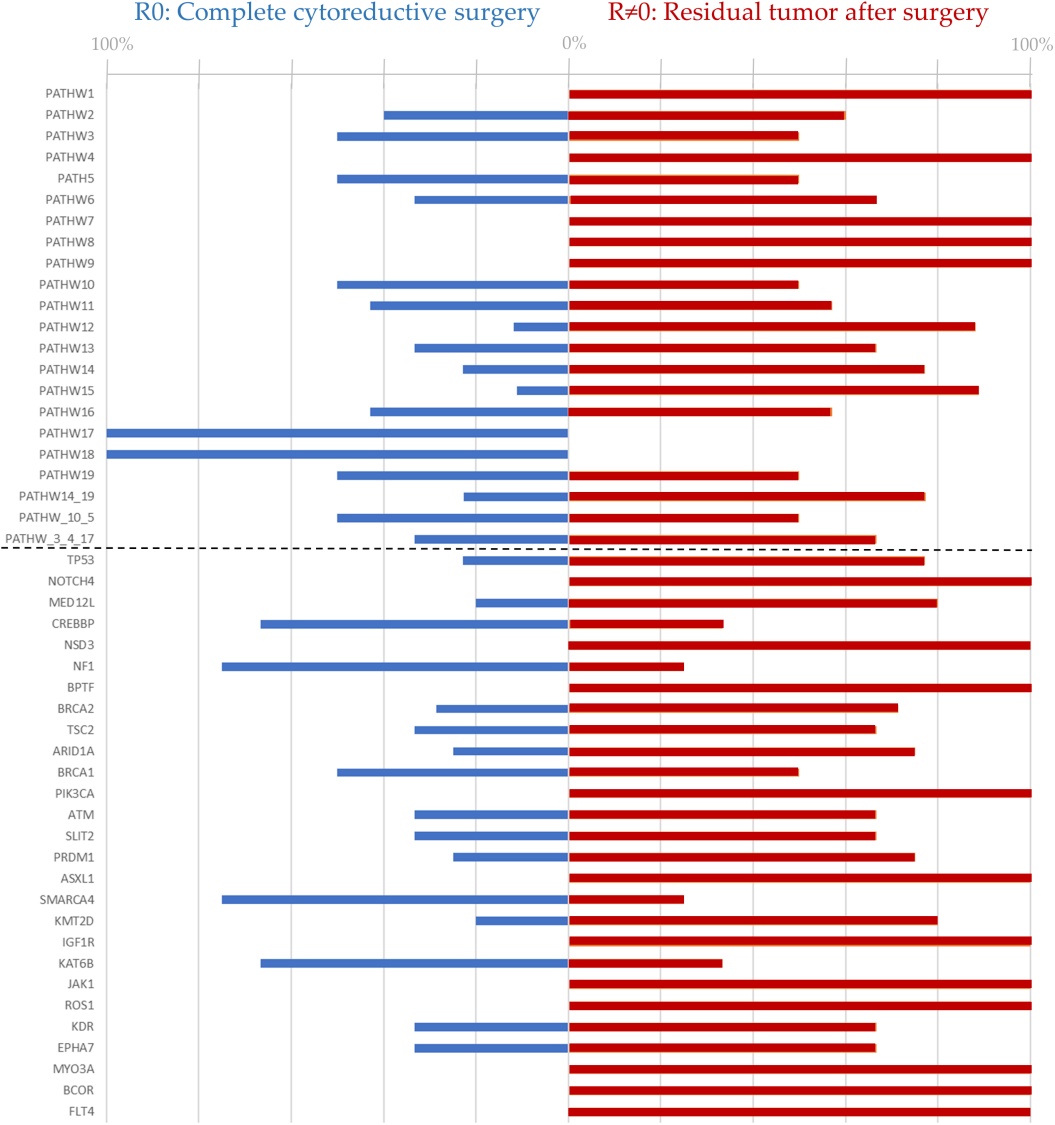
A bar graph illustrating the mutational status and pathway analysis results, according to surgical outcomes. Along the x‐axis, the genetic pathways are listed above the stipulated horizontal line, while the mutated genes are listed below. In the figure, the blue bars indicate genetic changes in the tumours of patients with complete cytoreductive surgery, while the red bars show which mutations can be found in the tumours of patients with residual tumours after surgery. Percentages of mutations are demonstrated along the x‐axis.

Overall, in the present sample set, 95% of the samples demonstrated at least one point mutation (Figure 2). The most frequently mutated genes were *TP53* (85.6%), followed by *MED12L* (5.20%), *BRCA2* (5.2%) and *KMT2D* (5.2%). Known germline *BRCA* mutations were included together with somatic *BRCA* mutations. Access to data for the germline *BRCA* was not included in the ethical approval statement for the project and was therefore unavailable. In a single‐gene model, we explored mutations occurring in more than 3% of the patients (*n* = 27) for associations with complete cytoreductive surgery. *NF1* and *SMARCA4* both had a significant association with complete cytoreductive surgery (*p* = 0.031). No significant associations to mutation status were found for optimal cytoreductive surgery (residual tumour <1 cm). We did not reveal any significant phenotypic traits for specific point mutations for residual tumours after surgery. However, some mutations were only found in patients with residual tumours (*NOTCH4*, *NSD3*, *BPTF*, *PIK3CA*, *ASXL1*, *IGF1R*, *JAK1*, *ROS1*, *MYO3A*, *FLT4*) (Figure 3). The most common mutations were missense, followed by frameshifts and deletions. Single nucleotide polymorphisms were the most common variant, and the most common single nucleotide variant class was C > T (Figures 2, 3).

### 3.3 Pathway analysis

Nineteen different pathways were analysed. (Table S3). Mutations in the chromatin remodelling SWI_SNF pathway (*p* = 0.036) and the histone H3K4 methylation pathway (*p* = 0.036) were significantly associated with complete cytoreductive surgery. The association increased when these pathways were combined (*p* = 0.002). The chromatin remodelling pathway mutations and histone H3K4 methylation mutations were found to be independent of co‐occurrence and not mutually exclusive (*p* = 0.27).

The presence of mutations in the chromatin remodelling pathway was found to be a prognostic factor for increased OS (*p* = 0.033, HR 2.691, 95% CI HR 1.082–6.696). Another observation was that mutations in the *P38* pathway and MSI pathway were only discovered in the R0 group, and mutations in the lineage maintenance transcription factors, ERK signalling and contact‐induced signalling pathways were only found in the R ≠ 0 group. The findings are not regarded as significant.

Mutations leading to a deficiency in the homologous recombination (HR) pathway represent a field of special interest due to the implementation of PARP inhibitors. Mutations previously classified as likely drivers involved in HR or other DNA damage repair pathways by predefined criteria were examined.^29^ In our cohort, 26.8% of the patients had a likely HRD and 16.5% had a strict HRD classification. In the HRD strict pathway, the OS was significantly longer (*p* = 0.044, OR 2.1, 95% CI 1.019–4.453), but subgroup analysis of surgical outcomes did not demonstrate a significant difference in OS. Patients with likely HRD had a longer time before recurrence (*p* = 0.007) and more advanced surgery (*p* = 0.018). Only four patients did receive a PARP inhibitor, and all as part of the relapse treatment.

### 3.4 Tumour mutational burden (TMB)

**FIGURE 5:**
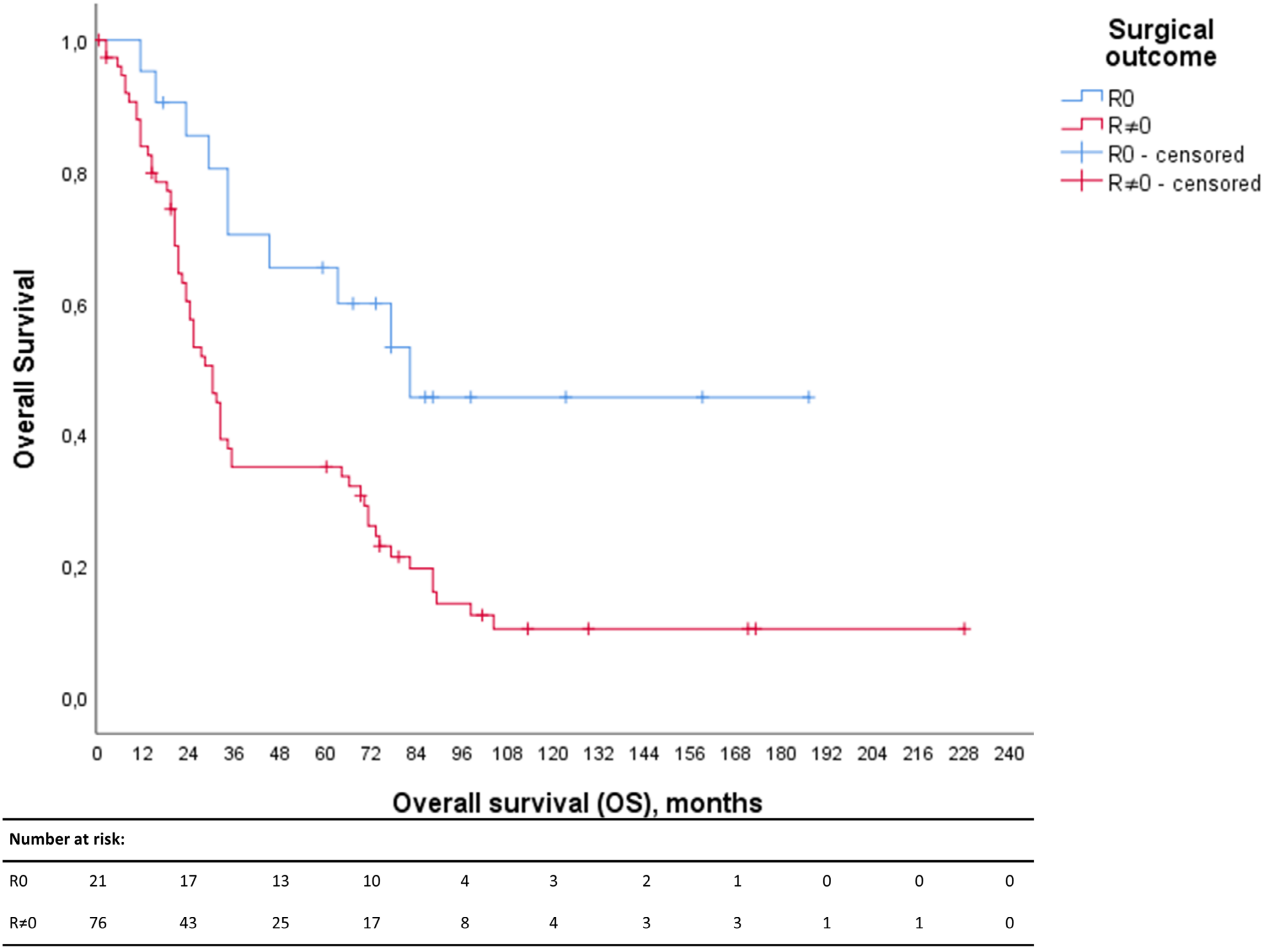
Cox survival curves of the HGSOC cohort. The patients are analysed separately based on whether they belong to the R0 or the R ≠ 0 cohorts. The curves show overall survival (OS) for patients with (the three upper plots) or without (the three lower plots) complete cytoreductive surgery. In all plots, the blue lines demonstrate survival curves for patients who do not harbour the specific mutational status while the red curves illustrate the OS for patients whose tumour carry a mutation in the genes: (A) Other DNA repair genes, (B) HRD genes, or (C) part of the chromatin remodelling pathway and/or histone methylation pathway. Below the survival curves a table is inserted showing the number of patients harbouring the specific mutational status in each cohort.

We compared the TMB in the R0 and R ≠ 0 group respectively (Figure 3). We found an increased TMB to be highly associated with complete cytoreductive surgery (*p* = 0.028, Figure 5). R0 (TMB 4.67, mean rank 60.8) and R ≠ 0 (TMB 3.62, mean rank 45.8).

### 3.5 Prediction model for complete cytoreductive surgery

In an exploratory approach, we selected single‐gene mutations associated with complete cytoreductive surgery with *p*‐values<0.2 as candidates to be included in an overall prediction model for primary surgical treatment. The best prediction model included five mutations: *CREBBP, NF1, BRCA1, SMARCA4* and *KAT6B*. Association analysis was performed with the strongest phenotypic traits: Preoperative clinical conclusion of stage and ascitic fluid >500 mL. Mutations in the histone methylation pathway were also included. Using the combination of genetic markers and phenotypic traits, we managed to develop a model that predicted the surgical outcome in 87.6% of the cases (positive predictive value [PPV] 0.52, negative predictive value [NPV] 0.97). A model that includes the most significant individual parameters (ascitic fluid <500 mL, preoperative clinical conclusion, histone H3K4 methylation pathway, chromatin remodelling pathway and *NF1*) demonstrated PPV 0.43 and NPV 0.96. If we include only the phenotypic parameters, the PPV is 0.29.

### 3.6 Survival analysis

**FIGURE 4:**
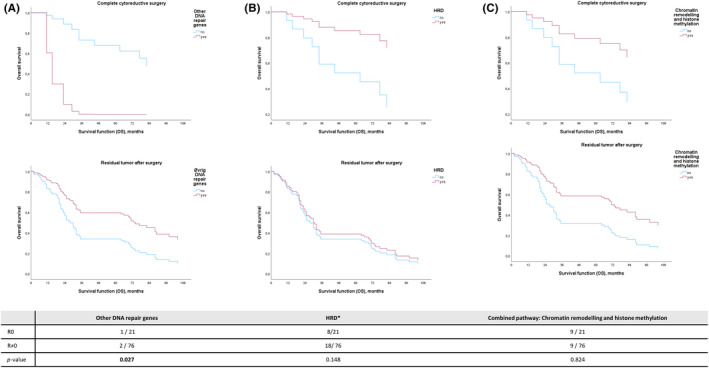
Kaplan–Meier survival analysis of the total HGSOC cohort stratified according to surgical outcome (R0 vs R ≠ 0). While the cumulative survival of the HGSOC cohort is depicted along the y‐axis, the duration of the overall survival in months is demonstrated along the x‐axis. Patients with complete surgical removal of tumour tissues (R0) are shown in blue, while the red line shows the patients with incomplete tumour tissue removal (R ≠ 0).

While patients with complete cytoreductive surgery demonstrated 110 months (76–143) OS, those patients who had residual tumour after surgery had an OS of 61 months (95% CI 45–78). Complete cytoreductive surgery was confirmed to be a prognostic factor for survival (*p* = 0.003; Figure 4).

To define mutations with an adverse outcome after surgery with residual tumour tissue, we performed multiple survival analysis of patients with specific gene and pathway mutations. In the group of genes that involve other DNA repair genes–such as *MLH1, MSH2* and *PARP10–*we observed a significant interaction (*p* = 0.027) between the covariates of resection rate and survival (Figure 5).

We also explored phenotypes as predictors of OS. Significant biomarkers for increased OS were ascitic fluid <500 mL (*p* = 0.037, HR 1.637, 95% CI 1.030–2.601), complete response after chemotherapy (*p* = 0.000, HR 2.188, 95% CI 1.660–2.884) and low ECOG score before surgical treatment (*p* = 0.006, HR 1.681, 95% CI 1.165–2.426). Links were identified between low albumin levels and low platelet counts and reduced OS (*p* = 0.005, HR 0.946, and *p* = 0.012, HR 0.636, respectively).

## 4 DISCUSSION

Although insight into the pathogenesis of HGSOC is growing, the effect of molecular heterogeneity on surgical outcome parameters has hardly been evaluated, either alone or in combination with phenotypic parameters, and is currently not included in existing treatment algorithms. Here, we have explored phenotypic traits and targeted DNA sequencing with the ambition of identifying biomarkers to improve the preoperative assessment. We have shown that patient characteristics, such as ascitic fluid volume and stage at diagnosis, affect the surgical outcome, and we have identified two methods of genetic profiling that reflect operability: mutational status including functional enrichment analysis and TMB assessment. In addition, we developed a combined preoperative risk stratification model, demonstrating that both clinical parameters and genetic profiling should be considered in the preoperative decision‐making process.

Primary cytoreductive surgery is the preferred treatment for women with advanced ovarian cancer. The aggressive surgical approach is unique within oncology, and no other malignant disease with disseminated spread has shown equal response to surgery. Multiple studies have identified that long‐term survival correlates with the completeness of cytoreduction.^3^, ^5^, ^31^ Patients in our cohort were selected for up‐front surgery based on the clinicians' choice of preoperative investigations, and the effect of complete cytoreduction on survival is in accordance with previous findings.^3^, ^5^, ^31^

Despite the introduction of advanced preoperative tools, the selection of suitable patients for ultra‐radical surgery is still challenging.^32^, ^33^, ^34^, ^35^ In this study, preoperatively established low volumes of ascitic fluid, lower CA125‐levels, higher platelet counts, and less advanced FIGO stage at diagnosis were all indicators, alone and combined, for complete cytoreduction. The surgical impact of ascitic volume has previously been demonstrated.^36^, ^37^, ^38^ Through the prospective documentation of tumour dissemination, according to the FIGO 2014 scoring system, we demonstrate the importance of phenotypic categorisation for personalised surgical treatment selection (Table 2). Furthermore, we show that phenotypic traits alone remain inadequate for identifying the patients eligible for surgical treatment.

There is an increasing number of studies assessing the effect of tumour biological characteristics on surgical outcome parameters,^39^, ^40^, ^41^ and different DNA alterations, gene expression and copy number signatures have been explored.^21^, ^25^, ^26^, ^39^, ^40^, ^41^ Besides *TP53*, *BRCA1* and *BRCA2* mutations, the most common recurring mutations in our study were *KMT2D*, *MED12L*, *ARID1A*, *PRDM1, SMARCA4, NF1* and *PIK3CA*. Our results demonstrate some overlap with gene mutations identified as impacting surgical outcomes in previous studies,^40^, ^42^, ^43^ but due to the differences in the methods used, comparisons cannot be made.

We launched a functional enrichment analysis in our study, which revealed that mutations in two pathways, alone or combined, were associated with complete cytoreductive surgery. Pathway analysis is performed to translate differently expressed gene mutations into meaningful biological events.^44^ The challenge in this type of analysis is that definition of the pathways, which evolves constantly due to new discoveries and accepted pathogenic variants,^45^ makes comparisons with prior findings difficult. We also could not identify any studies on HGSOC applying this definition, previously used in research on breast cancer.^46^

TMB has been widely implemented as a prognostic biomarker for immunotherapy response in cancer treatment, and it seems also to have a potential as a general prognostic indicator for outcomes in HGSOC, as an increased mutational rate has been linked to long‐term survival, lower FIGO stage and lower volume of residual tumours.^47^, ^48^, ^49^, ^50^, ^51^ Our findings demonstrated similar overall mutational rates as the The Cancer Genome Atlas (TCGA) based study.^21^ To our knowledge, TMB has not been explored as a marker to assist in the preoperative decision‐making process. Interestingly, we demonstrated that patients in the R0 group had significantly higher mutational rates than the R ≠ 0‐group and thereby that TMB seems to affect both the prognosis and the operability in this cohort.

Fagotti et al. found a PPV of 100% and an NPV of 70%, using a laparoscopic‐based model on optimal versus suboptimal debulking.^32^ Bristow et al. analysed a predictive index scoring system based on CT scans and demonstrated that an index score ≥4 had a PPV of 85% and an NPV of 100%.^52^ However, both of these methods use an archaic indicator of surgical success, allowing residual tumour ≥1 cm. Keunecke et al. validated an extended logistic regression model, including clinical factors and 93 genes, reduced by elastic‐net regularisation. This combined panel only reached an accuracy of 64.5%.^41^ Like Keunecke et al. we suggest a preoperative risk stratification model based on phenotypic traits and genotypic characteristics, maintaining that both surgical aggressiveness inherent and tumour biological characteristics influence operability and surgical outcome. Using logistic regression analysis, our best predictive model demonstrated surgical outcomes in 87.6% of cases. The most remarkable finding was that the PPV fell drastically when we only included phenotypic traits in our model, confirming the value of combining phenotypes and genotypes in an algorithm.

New strategies must be employed to improve survival rates for ovarian cancer. Tumour biological characteristics generate different metastatic patterns, which influence operability, but the paradox of the pre‐existing algorithms solely based on patient characteristics and stage is that even the subtypes characterised by worse outcomes or greater tumour mutation burden are likely to benefit from successful aggressive cytoreduction.^50^, ^53^, ^54^, ^55^ Complete surgery eliminates all macroscopic tumour mutations and hence equalises tumour biologic attributes. Phelps et al. showed that methylation of the *MYKL3* gene promoter region improved OS in patients with little or no residual disease, but without high methylation, the OS was identical to the R ≠ 0 group.^56^ Analogous to this, we found that mutations in the group of other DNA repair genes can be associated with OS (Figure 5). Other genetic changes, such as BRCA mutations and mutations in the HRD group, do not appear to significantly influence prognosis if residual tumour tissue is left behind. The analysis should be interpreted with caution due to the small sample size of patients in the R0 cohort and the limited occurrence of recurring mutations. These results are relevant for further investigations because they imply that both the preoperative evaluation and surgical aggressiveness should take into account the tumour mutational status.

There are several limitations to this study. The time frame is long (16 years) and the standard treatment regarding both surgical aggressiveness and adjuvant therapies has been adjusted during the period, which could affect the outcomes. To avoid the bias related to survival introduced by PARP inhibitors, we did not include any patients who received these drugs in the primary treatment setting. The definition of complete surgical cytoreduction has become stricter. To make the groups comparable across time, all operation records were examined and annotated by an experienced gynaecologist. Targeted DNA sequencing has many advantages, but there is the possibility of overlooking important drivers not included in the panel, and the panel prevented us from performing analysis for copy number alterations. Furthermore, the application of a targeted panel limits the applicability of the TMB estimates, and the results must be interpreted with caution. The low frequency of recurrent single mutations is a weakness in our prediction model, as illustrated by the *p*‐values of individual mutations in the multivariate model (Table 3), but the method could easily be replicated in other cohorts for validation. Further, the prediction model, although promising, is generated solely within our samples set. Validation in independent studies will be imperative for further development of the model.

**TABLE 3:** The results of logistic regression analysis of single‐gene mutations with *p*‐values<0.2 and selected pathways to include in a predictive model for complete cytoreductive surgery (R0).

Variables in Equation	*p*‐value	Standard Error	OR	95% CI for OR
Ascitic fluid: <500 mL	0.016	0.692	5.304	1.367–20.582
Clinical stage	0.087	0.476	2.262	0.889–5.754
Histone H3K4 methylation (KDM5A) pathway	0.177	0.899	0.297	0.051–1.730
CREBBP	0.207	1.632	0.129	0.005–3.107
NF1	0.179	1.656	0.108	0.004–2.774
BRCA1	0.080	1.184	0.126	0.012–1.285
SMARCA4	0.121	1.390	0.116	0.008–1.766
KAT6B	0.045	1.382	0.063	0.004–0.944

Abbreviations: OR; odds ratio, 95% CI; 95% confidence interval.

## 5 CONCLUSION

We demonstrate the potential benefit of including molecular biomarkers in the preoperative stratification of HGSOC patients. The strongest predictive biomarker for complete cytoreduction in our data was ascitic fluid volume < 500 mL, demonstrated in both the univariate and multivariate model. Combining predetermined phenotypic (ascitic fluid <500 mL, a predefined preoperative clinical conclusion) and genotypic (the histone H3K4 methylation (KDM5A) pathway, and the genes *CREBBP, NF1, BRCA1, SMARCA4* and *KAT6B*) pre‐operative biomarkers show potential for predicting complete cytoreduction in HGSOC patients. Although targeted DNA sequencing is increasingly available, the method has not yet been implemented in Norway in the clinical diagnostics of ovarian cancer. The fact that analysis of tumour genomic alterations is currently time‐consuming and costly, influences the implementation of genomic analysis as standard of care in cancer diagnostics. We propose that our risk prediction model, together with the TMB assessment, should be validated as a preoperative tool for estimation of complete cytoreductive success in new, well‐characterised clinical cohorts.

## AUTHOR CONTRIBUTIONS

**Cecilie Fredvik Torkildsen:** Conceptualization (equal); data curation (equal); formal analysis (lead); funding acquisition (equal); investigation (lead); methodology (equal); project administration (equal); software (equal); validation (lead); visualization (lead); writing – original draft (lead); writing – review and editing (lead). **Liv Cecilie Vestrheim Thomsen:** Conceptualization (supporting); data curation (supporting); formal analysis (supporting); funding acquisition (equal); investigation (equal); methodology (equal); project administration (equal); resources (equal); software (supporting); supervision (equal); validation (supporting); visualization (equal); writing – original draft (supporting); writing – review and editing (equal). **Ragnar Kvie Sande:** Conceptualization (supporting); data curation (supporting); formal analysis (supporting); funding acquisition (supporting); investigation (supporting); resources (supporting); software (supporting); supervision (supporting); validation (supporting); visualization (supporting); writing – original draft (supporting); writing – review and editing (supporting). **Camilla Krakstad:** Data curation (supporting); investigation (supporting); project administration (supporting); resources (supporting); writing – review and editing (supporting). **Ingunn Marie Stefansson:** Data curation (supporting); investigation (supporting); supervision (supporting); validation (equal); writing – review and editing (supporting). **Eva Karin Lamark:** Data curation (supporting); writing – review and editing (supporting). **Stian Knappskog:** Conceptualization (supporting); data curation (equal); formal analysis (equal); investigation (supporting); methodology (supporting); software (supporting); supervision (supporting); validation (supporting); visualization (equal); writing – review and editing (supporting). **Line Bjørge:** Conceptualization (lead); data curation (equal); formal analysis (equal); funding acquisition (equal); investigation (equal); methodology (equal); project administration (lead); software (equal); supervision (lead); validation (equal); visualization (supporting); writing – original draft (supporting); writing – review and editing (equal).

## FUNDING INFORMATION

This work was supported by grants from “The Western Norway Regional Health Authority” (Nos 912278, 91217, F‐12183/4800003665, F11635‐D11698), Stavanger University Hospital (No. 501809) and Folke Hermansen Foundation (No. 423204).

## CONFLICT OF INTEREST STATEMENT

Torkildsen reports personal fees from AstraZeneca and GlaxoSmithKline. Thomsen reports personal fees from Bayer, Eisai Co., and AstraZeneca, and Thomsen and Bjørge report financial support from AstraZeneca for a researcher‐initiated trial. The other authors declare no conflict of interest.

## ETHICAL APPROVAL STATEMENT

The study was conducted in accordance with the Declaration of Helsinki and approved by the Norwegian Regional Committees for Medical and Health Research Ethics (IDs 7226 and 8164).

## Supporting information


Data S1:

## Data Availability

The data that support the findings of this study are available on request from the corresponding author, pending project‐specific ethics approvals. The data are not publicly available due to ethical restrictions with respect to patient privacy.
