# Supplementary material for: Molecular and phenotypic characteristics influencing the degree of cytoreduction in high‐grade serous ovarian carcinomas
Source: Cancer Med. 2023 May 16;12(13):14183–95. doi: 10.1002/cam4.6085 (PMC10358208; doi:10.1002/cam4.6085)
Supplement: Supplementary file 1 — Data S1: [file CAM4-12-14183-s001.docx]

**Molecular and phenotypic characteristics influencing the degree of cytoreduction in high-grade serous ovarian carcinomas**

# Cecilie Fredvik Torkildsen

Centre for Cancer Biomarkers, Department of Clinical Science, University of Bergen, Norway

Department of Obstetrics and Gynecology, Stavanger University Hospital, Stavanger, Norway

cecilie.torkildsen@uib.no

# Liv Cecilie Vestrheim Thomsen

Centre for Cancer Biomarkers, Department of Clinical Science, University of Bergen, Norway

Department of Obstetrics and Gynecology, Haukeland University Hospital, Bergen, Norway

# Ragnar Kvie Sande

Department of Clinical Science, University of Bergen, Norway

Department of Obstetrics and Gynecology, Stavanger University Hospital, Stavanger, Norway

# Camilla Krakstad

Centre for Cancer Biomarkers CCBiO, Dep. of Clinical Science, University of Bergen, Norway

Department of Obstetrics and Gynecology, Haukeland University Hospital, Bergen, Norway

# Ingunn Stefansson

Department of Pathology, Haukeland University Hospital, Bergen, Norway

Centre for Cancer Biomarkers CCBiO, Dep. of Clinical Medicine, University of Bergen, Norway

**Eva Karin Lamark**

Department of Obstetrics and Gynecology, Haukeland University Hospital, Bergen, Norway

**Stian Knappskog**

Department of Clinical Science, University of Bergen, Norway

Department of Oncology, Haukeland University Hospital, Bergen, Norway

# Line Bjørge

Centre for Cancer Biomarkers CCBiO, Department of Clinical Science, University of Bergen, Norway

Department of Obstetrics and Gynecology, Haukeland University Hospital, Bergen, Norway

**SUPPLEMENTARY MATERIAL**

**Table S1** Descriptive statistics of the sequenced patients and the whole cohort of 477 HGSOC patients.

|  | **All HGSOC patients (n=477)** | **Sequenced (n=97)** |
| --- | --- | --- |
| Age in years (mean, 95% CI),(n) | 64 (63,1 – 65,1) (n=477) | 64 (62 – 66,3) (n=97) |
| Stage (FIGO 2014**) | (n=476) | (n=97) |
| *Stage 3* | *65,3%* | *77,3%* |
| *Stage 4* | *22%* | *22,7%* |
| Neoadjuvant chemotherapy (%) | 20,8 % (n=477) | 0% |
| Complete cytoreductive Surgery, % (n) | 35,3% (n=468) | 22,7% |
| BMI (m2/kg) at diagnosis (mean, 95% CI) | 25,4 (24,9–25,8) (n=470) | 25,3 (24,4–26,2) |
| CA125(kU/L) at diagnosis (mean, 95% CI) | 1528 (1200 – 1856) (n=462) | 1260 (929–1593) (n=94) |
| ASA^a^ score primary surgery (mean, 95% CI) | 2 (2,2–2,3) (n=456) | 2 (2,1 – 2,3) |
| ECOG^b^ score (mean, 95%CI) | 1 (0,7-0,8) (n=461) | 1 (0,6-0,9) (n=97) |
| Surgical complexity score^c^  (mean, 95% CI), (n) | 3 (3,0-3,3)(n=476) | 4 (3,2-4) |
| Clavien Dindo score^d^ (mean, 95% CI) (n) | 2 (1,7-1,9) (n=454) | 2 (1,5-2) |
| Peroperative ascitic fluid, ml (mean, 95% CI) (n) | 1412 (1167-1657) (n=281) | 1614 (1220-2007) |
| CA125 after surgery (mean, 95% CI) (n) | 557 (410 – 703), (n=396) | 778 (361-1195) (n=83) |
| Postoperative chemotherapy, % (n) | 91% (n=477) | 97,1% |
| Recurrence, % (n) | 84,7% (463) | 88% (n=83) |
| PFS / censored 31.12.22, mean (mnts), (95% CI) |  | 25,8 (19-33) |
| OS / censored 31.12.22, mean (mnts), (95% CI) | 54,5 (50-59) | 49,6 (41-59) |

**Table S2** (extended version). Subgroup classification into R0, R1 and R2.

| **Variable** | **P-value R0 vs ≥ R1** | **P value ≤ R1 vs R2** |
| --- | --- | --- |
| amFI score | 0.193 | 0.489 |
| Ploidy (diploid vs not diploid) | 0.204 | 1.000 |
| Categorical Survival: <3 years / > 5 years | **0.011** | 0.141 |
| Ascitic fluid: <500 ml | **0.002** | **0.001** |
| Smoking: ever vs current | 0.181 | 0.276 |
| Smoking: never vs ever | 0.447 | 0.296 |
| Previous abdominal surgery | 0.323 | 0.469 |
| Previous sterilization | 0.060 | 0.490 |
| Previous cancer disease | 0.728 | 1.000 |
| Previous PID | 1.000 | 0.373 |
| Tromboembolic disease | 0.685 | 1.000 |
| Diabetes mellitus | 0.582 | 0.656 |
| Cardiovascular disease | 0.510 | 1.000 |
| Lung disease | 0.643 | 1.000 |
| Clinical conclusion before surgery | **0.006** | **0.002** |
| ECOG score | 0.328 | **0.014** |
| Surgical complexity score | **0.000** | **0.001** |
| Complications (Clavien Dindo score) | 0.804 | 0.504 |
| Rounds of postoperative chemotherapy | 0.554 | 0.359 |
| Rubricated response evaluation | **0.000** | **0.035** |
| Ascites total volume | **0.008** | **0.000** |
| Stage of disease (FIGO) | **0.014** | **0.013** |
| Age | 0.324 | 0.363 |
| BMI | 0.674 | 0.131 |
| Number of children | 0.640 | 0.388 |
| Ca125 value at diagnosis | **0.022** | **0.012** |
| Platelets at diagnosis | **0.019** | **0.006** |
| LD at diagnosis | 0.234 | 0.739 |
| Time from surgery to chemotherapy | **0.026** | **0.002** |
| CA125 before initiation of chemotherapy | **0.000** | **0.000** |
| Progression-free survival | **0.006** | 0.053 |
| Overall survival | **0.009** | 0.060 |

Phenotypic markers analyzed in respect of complete cytoreductive surgery (R0). Analyses were also performed of optimal cytoreductive surgery (Includes residual tumor lesions >1cm) to demonstrate the difference of these groups. (Significant P-values highlighted)

Abbreviations: R0: Complete cytoreductive surgery, R1: Optimal cytoreductive surgery, tumor lesions ≤1 cm, R2: Suboptimal cytoreductive surgery, tumor lesions >1cm, amFI score: adjusted modified frailty index score, PID: Pelvic inflammatory disease, ECOG: Eastern Cooperative Oncology Group performance status,FIGO: International Federation of Gynecology and Obstetrics, (Fédération Internationale de Gynécologie et d’Obstétrique) classification of malignant ovarian tumors, BMI: Body Mass Index, CA125: Cancer antigen 125, LD: lactate dehydrogenase

**Table 3:** Overview of the pathways and their included genes

| **Pathways** | **Genes** |
| --- | --- |
| **Lineage maintenance transcription factors** | \| GATA3 \| \| --- \| \| ESR1 \| \| RUNX1 \| \| FOXA1 \| |
| **Estrogen receptor transcriptional activity** | \| ESR1 \| \| --- \| \| NCOA2 \| \| NCOA3 \| \| EP300 \| \| FOXA1 \| \| GATA3 \| |
| **cJUN signalling** | \| MAP3K1 \| \| --- \| \| MAP2K4 \| \| MAP2K7 \| \| MAP3K5 \| \| MAP3K9 \| \| JUN \| |
| **ERK signalling** | \| MAP3K1 \| \| --- \| \| BRAF \| \| RAF1 \| \| MAPK1 \| \| MAP2K1 \| \| MAP2K2 \| \| MAP3K8 \| |
| **Histone H3K4 methylation** | \| MLL3 \| \| --- \| \| MLL2 \| \| MLL \| \| KDM5A \| \| KDM5C \| |
| **Wnt beta catenin** | \| CDH1 \| \| --- \| \| CTNNB1 \| \| APC \| \| AXIN1 \| |
| **Contact induced signalling** | \| CDH1 \| \| --- \| \| NF2 \| \| EGFR \| |
| **Ecadherin transcriptional regulation** | \| TBX3 \| \| --- \| \| CDH1 \| \| FOXA1 \| \| GATA3 \| |
| **Notch signalling** | \| NOTCH1 \| \| --- \| \| NOTCH1 \| \| NOTCH2 \| \| NOTCH3 \| \| NOTCH4 \| \| FBXW7 \| |
| **Chromatin remodelling SWI_SNF** | \| ARID1A \| \| --- \| \| ARID1B \| \| SMARCA4 \| \| ARID2 \| \| SMARCB1 \| |
| **RAS GTPase** | \| NF1 \| \| --- \| \| HRAS \| \| KRAS \| \| NRAS \| |
| **Receptor tyrosine kinase upstream of RAS** | \| EGFR \| \| --- \| \| ERBB2 \| \| ERBB3 \| \| ERBB4 \| \| IGF1R \| \| IGF2R \| \| FGFR1 \| \| FGFR2 \| \| FGFR3 \| \| FGFR4 \| \| PDGFRA \| \| PDGFRB \| \| NGFR \| \| KIT \| \| FLT1 \| \| FLT4 \| \| KDR \| \| MET \| \| INSR \| |
| **Fanconi anemia Homologous recombination** | \| BRCA1 \| \| --- \| \| BRCA2 \| \| BRIP1 \| \| PALB2 \| \| FANCA \| \| FANCC \| \| FANCD2 \| \| FANCE \| \| FANCF \| \| FANCG \| \| BLM \| |
| **DNA damage response** | \| TP53 \| \| --- \| \| ATM \| \| CHEK2 \| |
| **PI3 kinase** | \| PIK3CA \| \| --- \| \| PIK3R1 \| \| PIK3R2 \| \| AKT1 \| \| AKT2 \| \| AKT3 \| \| PTEN \| |
| **MTOR** | \| MTOR \| \| --- \| \| TSC2 \| \| TSC1 \| \| RPTOR \| \| RICTOR \| \| RPS6KB1 \| |
| **p38** | \| MAP3K5 \| \| --- \| \| MAP2K6 \| |
| **Microsatellite instability** | \| MSH2 \| \| --- \| \| MSH6 \| \| SETD2 \| |
| **Retinoblastoma pathway** | \| RB1 \| \| --- \| \| CDK4 \| \| CCNE1 \| \| E2F3 \| \| CCND1 \| |
|  |  |
|  |  |


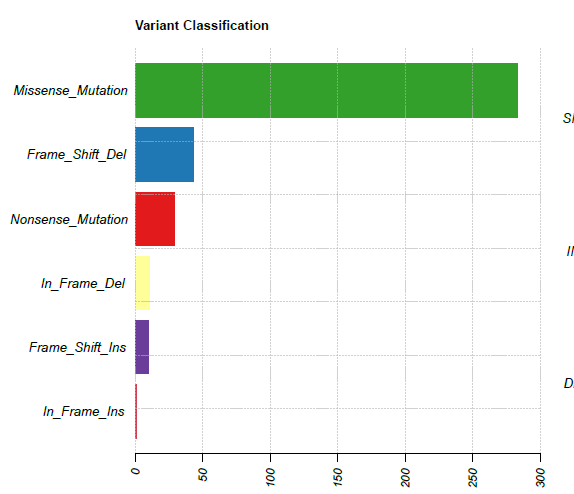

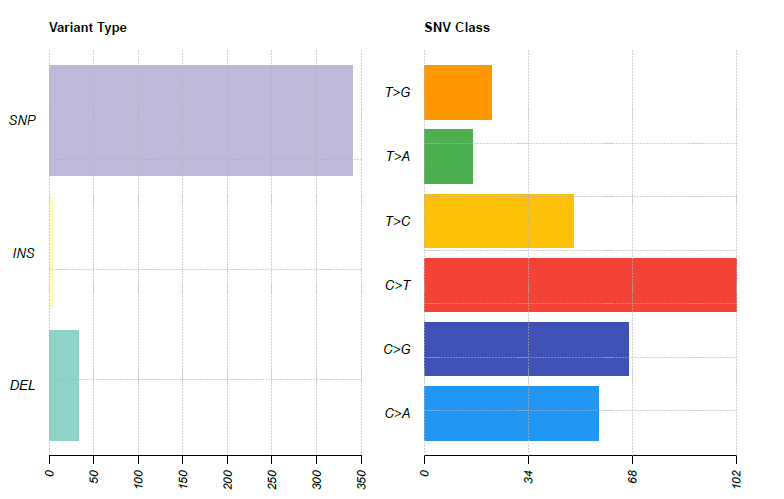

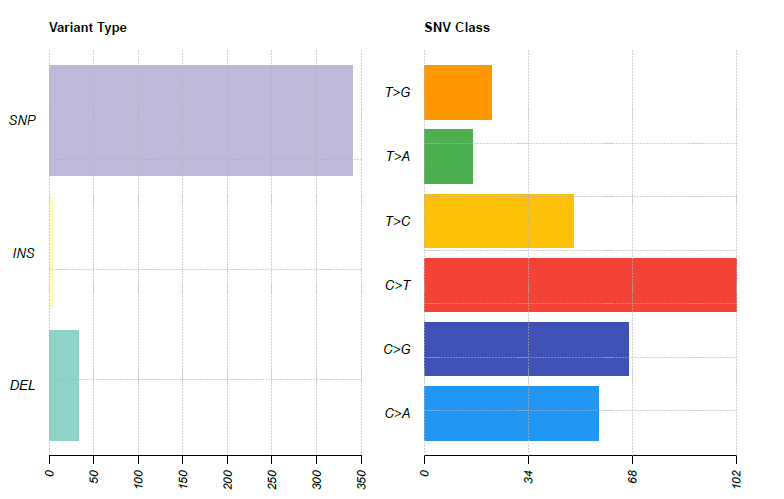


**Figure S1** Graphical illustration of variant classification, variant type and SNV class of mutations.


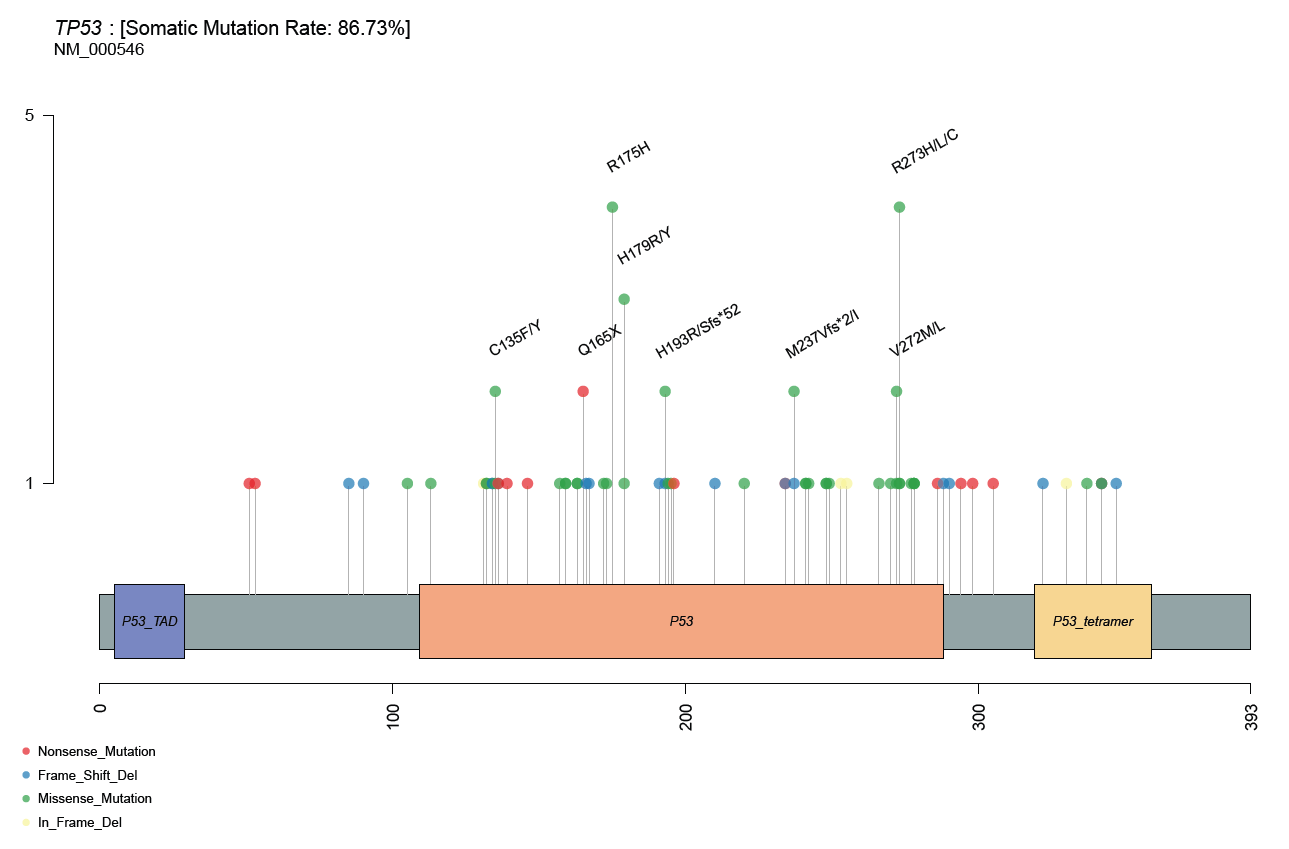


**Figure S2** Graphical illustration of the TP53 mutations occurring in the study.
